# Occurrence of status epilepticus in persons with epilepsy is determined by sex, epilepsy classification, and etiology: a single center cohort study

**DOI:** 10.1007/s00415-021-10600-y

**Published:** 2021-05-21

**Authors:** Lisa Langenbruch, Christine Strippel, Dennis Görlich, Christian E. Elger, Gabriel Möddel, Sven G. Meuth, Christoph Kellinghaus, Heinz Wiendl, Stjepana Kovac

**Affiliations:** 1 Department of Neurology with Institute of Translational Neurology, University of Münster, University Hospital Münster, Albert-Schweitzer-Campus 1, Gebäude A1, 48149 Münster, Germany; 2 Institute of Biostatistics and Clinical Research, University of Münster, Münster, Germany; 3 Department of Neurology, University of Düsseldorf, Düsseldorf, Germany; 4 Department of Neurology, Klinikum Osnabrück, Osnabrück, Germany

**Keywords:** Comorbidity, Seizure, Perception, Rescue medication

## Abstract

**Background:**

Status epilepticus (SE) can occur in persons with or without epilepsy and is associated with high morbidity and mortality.

**Methods:**

This survey aimed to record self-reported frequency of SE in persons with epilepsy, its association with clinical characteristics and patient level of information on SE and rescue medication. 251 persons with epilepsy at a tertiary epilepsy center were included in the study.

**Results:**

87 (35%) had a history of SE defined as seizure duration of more than 5 min. These patients were less likely to be seizure-free, and had a higher number of present and past anti-seizure medication. Female sex, cognitive disability, younger age at epilepsy onset, defined epilepsy etiology, and focal epilepsy were associated with a history of SE. On Cox regression analysis, female sex, defined etiology and focal classification remained significant. 67% stated that they had information about prolonged seizures, and 75% knew about rescue medication. 85% found it desirable to receive information about SE at the time of initial diagnosis of epilepsy, but only 16% had been offered such information at the time.

**Conclusion:**

SE is frequent among persons with epilepsy and there remain unmet needs regarding patient education.

## Introduction

Status epilepticus (SE) can lead to long-term neurological sequelae or death and is associated with increased rates of drug-refractory epilepsy with reduced quality of life [1, 2]. Most epidemiological studies focus on SE in the general population, a heterogeneous cohort consisting of > 50% of persons without preexisting epilepsy [3–9]. These studies applied a cut-off of 30 min to define SE. The current ILAE operational definition of SE allows diagnosis of (convulsive) SE after 5 min [10]. Beyond this point in time, rescue medication is often necessary to disrupt seizure activity. In an outpatient setting, the physician needs to estimate the patients’ risk of SE and decide whether patients are supplied with rescue medication. Surprisingly, knowledge on factors associated with SE in persons with preexisting epilepsy is scarce. Prescription of rescue medication is often limited to those who already suffered an episode of SE. To address these gaps, this study aimed to answer the following questions: how many patients with epilepsy report a history of SE with a cut-off at 5 min? Which demographic or clinical characteristics are associated with a history of SE? What is the level of information about SE in a tertiary epilepsy clinic?

## Materials and methods

We conducted a survey including 251 consecutive patients with epilepsy at the outpatient (95%) and inpatient (5%) epilepsy clinic of the University Hospital Münster, Germany. Patients were asked to participate in a structured interview if they had any epilepsy diagnosis, and they or their legal guardian gave consent. The interview was carried out with the help of a standardized questionnaire. Primary endpoints were the frequency of SE and patient level of information on SE and rescue medication. Secondary endpoints were demographic and clinical characteristics and their association with a life-time history of SE.

Patients with newly diagnosed epilepsy, i.e. if an epilepsy diagnosis was made at the current visit, were not included. The small share of patients recruited from the inpatient epilepsy ward was included for reason of availability, but not due to SE-related admission. All other patients were recruited consecutively from the outpatient epilepsy clinic. For evaluation of life-time history of SE, a cut-off of 5 min of seizure duration was applied regardless of seizure semiology. Applying this cut-off is technically only appropriate for bilateral tonic–clonic seizures, as focal seizures frequently last longer [11]. We still chose a 5 min cut-off as we deemed it unlikely to achieve differentiation in the patient interview. For seizures with motor onset, participants were asked to judge only the duration of motor phenomena to exclude postictal states. In few patients with known idiopathic generalized epilepsies, absence status was included. Seizure semiology and duration were judged according to information of family members or caregivers. Further time points (30 and 60 min and 24 h) were examined as we deemed it likely achieve reliable differentiation from ongoing seizure activity and postictal states at time points that are out of the usual range of postictal states. The interviewer took care to discriminate epileptic and non-epileptic seizures, if applicable, again taking into account the patient’s and family members’ reports. Questions on perception of SE were answered by the patient, or by the legal guardian, if the patient was not able to express an opinion. All available information on case history, imaging, and EEG data were considered regarding seizure and epilepsy classification. Patients at our center routinely undergo high-quality imaging and video-EEG-monitoring if routine EEG and imaging are not conclusive in ascertaining epilepsy etiology and classification.

Statistical analysis and figure preparation were performed with SPSS (IBM corp., version 25.0). For group comparisons, Mann–Whitney U or Chi-Square/Fisher’s exact test was applied. Because some parameters yielded multiple subgroups with only small numbers, we then chose to compare single subgroups to the sum of all other subgroups. This applies to classification (subgroups focal, generalized, focal and generalized, and unknown) and etiology (genetic, tumor, vascular, dysplasia, infectious, autoimmune, hippocampal sclerosis, other defined etiology, unknown etiology), here Chi-Square/Fisher’s exact test was used.

Multivariable logistic regression models were applied to estimate the risk for a SE event for relevant potential risk factors: sex, age at epilepsy onset, epilepsy classification, epilepsy etiology, and cognitive disability. The multivariable model was estimated as one unique model comprehensive of all risk factors. All variables were categorical. Age at epilepsy onset was defined as infancy up to one year, two to nine years, or 10 years and above. Epilepsy classification was defined as focal epilepsy or non-focal epilepsy including unknown classification. Epilepsy etiology was defined as defined versus unknown etiology. Odds ratios (OR) will be reported. Survival analysis was performed for the event-free survival (EFS) times. EFS is defined as time from epilepsy onset to SE. Patients without event were censored at the date of the survey. Survival curves are shown as Kaplan–Meier curves and numbers at risk. For group comparisons, two-sided log-rank tests were applied. Furthermore, multivariable Cox regression models were fitted to assess hazard ratios (HR) for the five above-mentioned categorical variables. For logistic and Cox regression models, Wald test *p*-values and 95% confidence intervals (CI) will be reported.

## Results

Demographic and clinical characteristics are presented in Table 1. 35% of patients had experienced at least one episode of SE. 6% of all patients experienced SE as the initial seizure, but a first SE was reported as late as 48 years after epilepsy onset. 78% of all first SE episodes were bilateral tonic–clonic seizures. In most patients (69%), precipitating factors were not known (Table 2). 72% reported SE duration of < 30 min. 20% received rescue medication before arrival of medical personnel, and 59% by medical personnel, but general anaesthesia was rarely required (8%).

**Table 1:** Demographic and clinical data of the total group and comparison of the subgroups with and without history of SE

	All patients	Subgroup without history of SE (*n* = 164)	Subgroup with history of SE (*n* = 87)	*p*
History of SE (*n*, %)	87 (34.7)			
History of > 1 episode of SE (*n*, %)			48 (55.8)	
Women (*n*, %)	133 (53)	78 (47.6)	55 (63.2)	0.02^b^
Age in years (median, IQR)	36 (26–53)	36 (26–55)	37 (25–47)	0.65^a^
Age at first seizure (median, IQR)	16 (7–33)	19 (10–35)	11 (2–24)	< 0.001^a^
Time since epilepsy onset in years (median, IQR)	18 (7–27)	15 (6–25)	21 (13–29)	0.001^a^
History of febrile seizures (*n*, %)	26 (10.7)	19 (11.8)	7 (8.6)	0.45^b^
History of bilateral tonic clonic seizures (*n*, %)	206 (82.1)	131 (79.9)	75 (86.2)	0.21^b^
Number of present ASM (median, IQR)	2 (1–2)	1.5 (1–2)	2 (1–3)	< 0.001^a^
Number of past ASM (median, IQR)	2 (1–4)	1 (0–3)	3 (1–5)	< 0.001^a^
Seizure-free for > 1 year (*n*, %)	106 (42.2)	79 (48.2)	27 (31.0)	0.009^b^
Classification of epilepsy (*n*, %)				
Generalized epilepsy	38 (15.1)	29 (17.7)	9 (10.3)	
Focal epilepsy	186 (74.1)	117 (71.3)	69 (79.3)	
Generalized and focal	11 (4.4)	6 (3.7)	5 (5.7)	
Unknown	16 (6.4)	12 (7.3)	4 (4.6)	
Etiology of epilepsy (*n*, %)				
Genetic	48 (19.1)	30 (18.3)	18 (20.7)	
Tumor	23 (9.2)	16 (9.8)	7 (8.0)	
Vascular	22 (8.8)	13 (7.9)	9 (10.3)	
Dysplasia	20 (8.0)	9 (5.5)	11 (12.6)	
Hippocampal sclerosis	15 (6.0)	12 (7.3)	3 (3.4)	
Autoimmune	13 (5.2)	10 (6.1)	3 (3.4)	
Infectious	10 (4.0)	3 (1.8)	7 (8.0)	
Traumatic	7 (2.8)	5 (3.0)	2 (2.3)	
Other specific etiology	6 (2.4)	1 (0.6)	5 (5.7)	
Unknown	87 (34.7)	65 (39.6)	22 (25.3)	
Comorbidities (past and present) (*n*, %)				
Major depression	83 (33.1)	53 (32.3)	30 (34.5)	0.73^b^
Migraine	53 (21.1)	32 (19.5)	21 (24.1)	0.39^b^
Arterial hypertension	48 (19.1)	27 (16.5)	21 (24.1)	0.14^b^
Cognitive disability	44 (17.5)	23 (14.0)	21 (24.1)	0.045^b^
Intracranial neoplasm	28 (11.2)	17 (10.4)	11 (12.6)	0.59^b^
Pulmonary disease	22 (8.8)	12 (7.3)	10 (11.5)	0.27^b^
Ischemic stroke	20 (8.0)	13 (7.9)	7 (8.0)	0.97^b^
Extracranial neoplasm	15 (6.0)	11 (6.7)	4 (4.6)	0.50^b^
Substance abuse	14 (5.6)	7 (4.3)	7 (8.0)	0.22^b^
Psychogenic seizures	12 (4.8)	4 (2.4)	8 (9.2)	0.03^c^
Eating disorder	10 (4.0)	7 (4.3)	3 (3.4)	1.0^c^
Intracranial hemorrhage	10 (4.0)	6 (3.7)	4 (4.6)	0.74^c^
Knowledge of SE (*n*, %)	167 (66.5)	98 (59.8)	69 (79.3)	0.001^b^
Knowledge of rescue medication (*n*, %)	189 (75.3)	114 (69.5)	75 (86.2)	0.004^b^
Information on SE desired at initial diagnosis (*n*, %)	209 (85.3)	132 (81.5)	77 (92.8)	0.02^b^
Information on SE received at initial diagnosis (*n*, %)	31 (15.7)	16 (12.2)	15 (22.7)	0.13^b^

Some data were missing for individual patients, and percentages were calculated for patients with available data only

*SE* status epilepticus; *IQR* interquartile range

^a^Mann–Whitney U

^b^Pearson Chi-Square

^c^Fisher’s exact test (2-sided)

**Table 2:** Clinical data of first and last episodes of SE; data given as* n* (%) unless otherwise indicated

	*n*	First SE	*n*	Last SE for patients with > 1 SE
Bilateral tonic–clonic SE	81	63 (77.8)	44	20 (45.5)
SE as first seizure	86	16 (18.6) 6.4% of total cohort		
Time from seizure onset to first SE in years (median, IQR)	87	6 (0–20)		
Cause of SE	74		39	
Fever/infection		5 (6.8)		2 (5.1)
ASM change		4 (5.4)		9 (23.1)
Sleep deprivation		4 (5.4)		1 (2.6)
Acute cerebral damage		4 (5.4)		0
Substance withdrawal		2 (2.7)		1 (2.6)
Missed ASM intake		1 (1.4)		0
Specific other cause		3 (4.1)		0
No identifiable cause		51 (68.9)		26 (66.7)
Seizures in the last three months before the SE	70	40 (57.1)	36	30 (83.3)
Duration of SE	76		42	
5–29 min		55 (72.4)		28 (66.7)
30–59 min		3 (3.9)		4 (9.5)
60 min–24 h		15 (19.7)		8 (19.0)
> 24 h		3 (3.9)		2 (4.8)
Rescue medication administered by relatives	77	15 (19.5)	43	18 (41.9)
Rescue medication administered by medical personnel	68	40 (58.8)	39	16 (35.6)
General anaesthesia	79	6 (7.6)	44	1 (2.3)
Number of current ASM at onset of SE	71		38	
0		31 (43.7)		3 (7.9)
1		22 (31.0)		12 (31.6)
2		14 (19.7)		10 (26.3)
3		3 (4.2)		8 (21.1)
4		1 (1.4)		1 (2.6)
> 4		0		4 (10.5)
Modification of ASM regime because of SE	71	54 (76.1)	40	26 (65.0)

*SE* status epilepticus; *ASM* anti-seizure medication; IQR, interquartile range

**Fig. 1:**
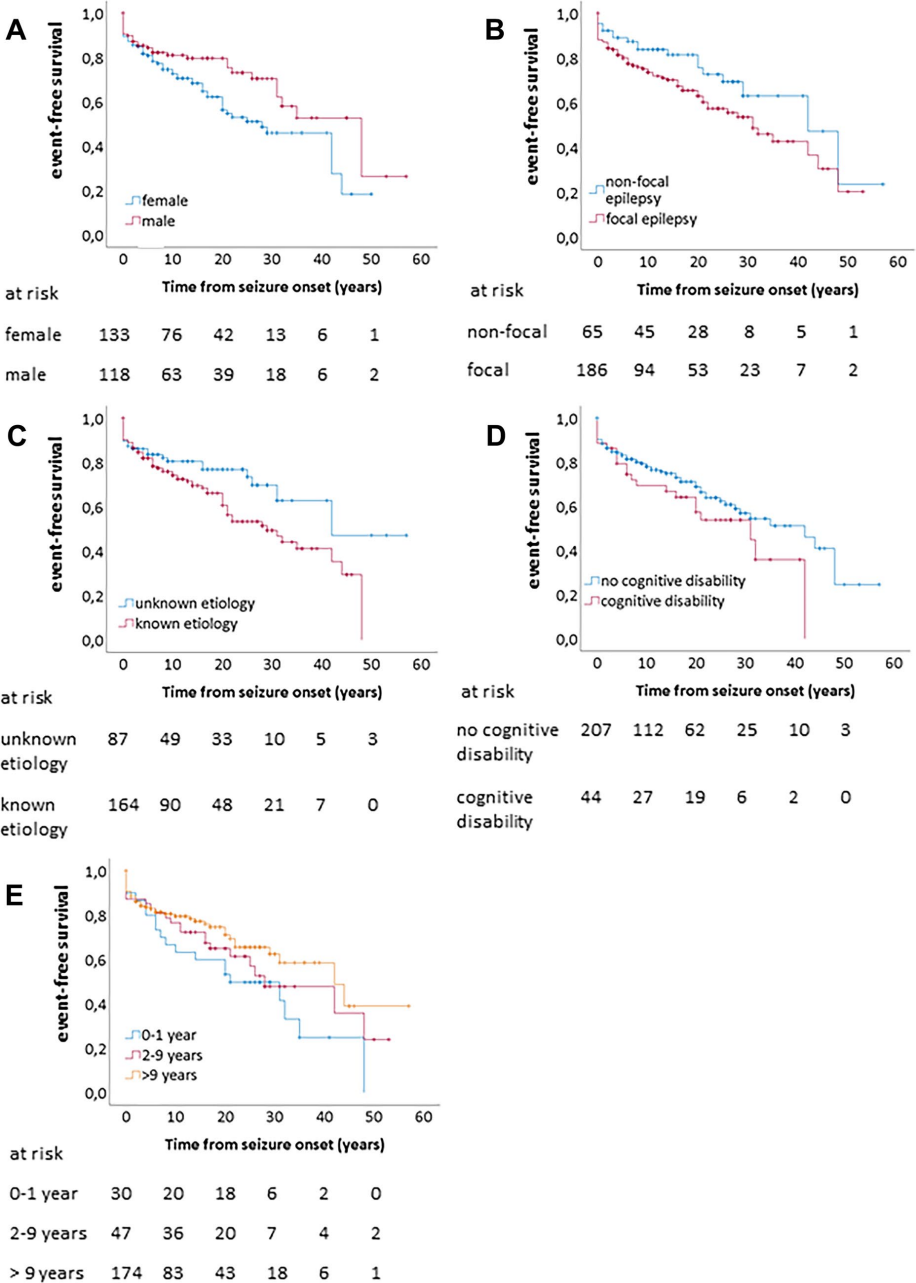
Kaplan–Meier plots of event-free survival, i.e. without a status epilepticus, after epilepsy onset. “At risk” indicates the number of remaining cases

Patients with a history of SE were less likely to have been seizure-free for the last year before the interview, and their number of present and past anti-seizure medications (ASM) was higher (Table 1). They were also younger at the first seizure and had a longer duration of epilepsy. SE was particularly frequent in patients with epilepsy onset in infancy (63%) and at 2–9 years (47%). With later epilepsy onset, SE was reported in 21–31%. Women were more frequent in the group with SE history (Fig. 1). There was an increased share of patients with focal epilepsy in the SE group compared with other and unknown classification taken together. If comparing specific etiologies with the sum of all other etiologies, patients with epilepsy of unknown etiology were significantly less frequent and patients with epilepsy due to dysplasia/migration disorders or CNS infection more frequent in the SE group. Of the comorbidities, only cognitive disability and psychogenic seizures were more common in the SE group.

We performed multiple logistic regression using the categorical variables sex, cognitive disability, age at epilepsy onset (0–1 years vs. 2–9 years vs. 10 years or older), classification of epilepsy (focal vs. other or unknown) and etiology (unknown vs. known incl. classic idiopathic generalized epilepsy syndromes). In this model, female sex, younger age at onset, focal classification and defined etiology remained significantly associated with SE (Table 3). We speculated that time to event from diagnosis of epilepsy might account for group differences. Taking into account time to first SE from epilepsy onset in Cox regression, age at onset did not remain significant, while sex, focal classification and defined etiology were still associated with a higher frequency of SE.

**Table 3:** Logistic and Cox regression analysis on factors associated with the occurrence of status epilepticus

	Multivariate logistic regression			Cox regression		
	*p*	OR	CI	*p*	HR	CI
Female sex	0.02	1.95	1.10–3.46	0.01	1.76	1.13–2.75
Age at seizure onset infancy to 1 year	0.001	10.15	2.70–38.18	0.15	1.72	0.82–3.58
2–9 years	0.002	3.35	1.57–7.16	0.26	1.38	0.79–2.43
Focal classification	0.03	2.17	1.09–4.31	0.02	1.85	1.09–3.14
Defined etiology	0.02	2.17	1.16–4.08	0.04	1.71	1.03–2.85
Cognitive disability	0.14	0.43	0.14–1.31	0.99	1.00	0.51–1.99

*OR* odds ratio; *CI* confidence interval; *HR* hazard ratio

67% of the patients had heard of prolonged seizures and 75% about rescue medication. 85% expressed the wish of receiving education about SE at initial diagnosis of epilepsy, while retrospectively 16% stated that it had in fact been discussed with them at the time. In patients with a history of SE, knowledge about rescue medication was significantly more common, and patients expressed more often the retrospective desire of discussion at initial diagnosis.

## Discussion

Prolonged seizures of > 5 min duration were reported by 35% of this population of persons with epilepsy. In the few long-term cohort studies, 22 to 27% of persons with epilepsy at a residential care centre as a highly selected epilepsy cohort had an episode of SE (> 30 min) in their lifetime [12, 13]. In population or register studies of the general population 12 to 50% of SE occurred in persons with preexisting epilepsy [3–9]. All these studies apply a cut-off of 30 min duration and do not account for prolonged seizures which may have ended spontaneously or received treatment to prevent longer duration. Some patients will have been diagnosed with epilepsy after a first-ever SE, so the percentage of SE associated with epilepsy may be higher than indicated. Our cohort reported SE as the first seizure in 19% of the subgroup with a history of SE, or 6% of the total group. Other authors found that a first-ever SE resulted in a diagnosis of epilepsy in 35% of SE associated with epilepsy [5]. In a cohort of childhood-onset epilepsies, SE occurred before the onset of epilepsy or resulted in a diagnosis in 73% of SE episodes [13]. This variability indicates age-specific incidences. In addition, prolonged febrile seizures and higher rates of new onset refractory SE may explain this relatively high number [14]. The influence of patient age is affirmed by our finding of higher rates of SE in patients with epilepsy onset at very young age, although age did not remain significant in Cox regression analysis.

Epilepsy of unknown cause was less frequently associated with SE than structural or genetic epilepsy as demonstrated in our study with 25 vs. 40%, or in the aforementioned cohort of childhood-onset epilepsy with 22 vs. 39% [13]. This may indicate more profound network alteration in lesional epilepsies, although this remains speculative. Patients at our center routinely undergo high-quality imaging and video-EEG-Monitoring if the cause of the epilepsy is unclear, so we deemed it unlikely that we overestimated the proportion of patients with epilepsy of unknown cause. Additionally, the proportion of patients with unknown etiology corresponds to epidemiological studies [15]. Corresponding to the trend of higher incidences of drug-resistant epilepsy in focal versus generalized epilepsies, focal origin was associated with a higher frequency of SE in the present study [1].

In our study, female sex remained significantly associated with a history of SE in logistic and Cox regression analysis. It remains to be seen whether this reflects a true risk constellation or a reporting bias. Some population-based studies found a higher incidence of SE in men which may be attributed to higher incidences of risk constellations such as cerebrovascular diseases, especially in adult populations [5, 6, 8]. There were no sex differences in children with or without association with epilepsy [3]. However, SE incidence was reported to be higher in men in all age groups in two studies with particularly high ratios in young children and the elderly in one study and in middle age in the other [5, 16]. Two studies in an Italian population showed the contrary with especially high SE incidences in older women [17, 18]. This was explained by a higher risk of dementia and more severe disease course in acute cerebrovascular disorders in women. Both factors are unlikely to play a role in our cohort, since the median age was relatively low and SE frequency was higher in women in all age groups (data not shown). All of these studies examined SE epidemiology in the general population which makes comparison with our cohort of epilepsy patients difficult.

Psychogenic non-epileptic seizures as a comorbidity were more common in the group with a history of SE, although absolute numbers were small. The treating physician took care to differentiate psychogenic and epileptic seizures in the interview to reduce the risk of falsely including a prolonged psychogenic seizure as SE. It would be an interesting question for further studies whether psychogenic seizures are indeed associated with a more severe disease course or other complications of epilepsy.

The survey showed a lower rate of seizure-free patients in the subgroup with SE. This is in line with a meta-analysis of risk factors for drug-resistant epilepsy [1]. Low ASM levels are commonly named as the most frequent etiology of SE in persons with epilepsy [4, 19]. In this study, patients rarely (in only 7%) recalled changes of ASM regime or missed doses as provocative factors. Most patients (69%) did not recall any specific catalytic circumstance. The discrepancy may be explained by patient recruitment, as most population-based studies recruit hospitalized SE patients. SE due to low ASM levels usually requires loading with ASM and may be overrepresented among hospitalized patients.

Only two-thirds of the patients expressed knowledge about prolonged seizures and SE. With SE being a potentially hazardous complication of epilepsy and rescue medication being available, patient education needs to be improved. Surprisingly, even 21% of those with a history of a prolonged seizure seemed not to be aware of the significance. A large majority voted for a discussion of SE at the initial epilepsy diagnosis. This desire has clearly not been met for most patients. Certainly, the discussion of the disease and its medical, social and emotional consequences at the initial diagnosis has to be conducted with care. Mentioning life-threatening complications like SE can be challenging and, of course, should be undertaken cautiously. On the other hand, discussing rescue medication may help the patient and family feel more confident. Knowledge about rescue medication for prolonged seizures was—unsurprisingly—more common in the group with SE. Still, 14% did not know about its availability.

Obviously, the study is limited by the reliance on patient and family statements. Seizure duration is generally difficult to judge for an observer and inferior to video-EEG-documentation. Including seizures with a cut-off of 5 min seizure duration may overestimate SE occurrence. Restricting the data to patients in a tertiary epilepsy center may also lead to a strong sampling bias towards patients with refractory epilepsy. Another inherent bias of a survey is that patients who died from SE cannot be included. This may lead to an underestimation of SE incidence and an exclusion of cases with more severe disease course. This may also explain the low rate of refractory SE or general anaesthesia. The survey may have missed cases of non-convulsive SE following seizures with motor onset, as we recorded only the duration of motor phenomena in the beginning of a seizure to exclude patients with prolonged postictal states and thereby avoid overestimation of SE frequency.

## Conclusion

SE is a frequent complication in persons with epilepsy, especially in women, patients with focal epilepsy, defined etiology, very young age at epilepsy onset, or cognitive disability. Most patients’ desire information about SE even early in the disease course, and patient level of knowledge about this complication and its treatment should be carefully evaluated by the treating neurologist.

## Data Availability

The authors have full access to all data. Data are not shared for ethical reasons.
